# The Effects of Using Chemicals to Remove Slime from African Giant Land Snails Flesh during Processing on Some Nutritional and Biochemical Parameters

**DOI:** 10.1155/2021/6691609

**Published:** 2021-04-21

**Authors:** Agbor Esther Etengeneng, Lamye Glory Moh, Suffo Kamela Arnaud Landry

**Affiliations:** ^1^Department of Biochemistry, Faculty of Science, University of Dschang, P.O. Box 67, Dschang, Cameroon; ^2^Department of Biomedical Sciences, Faculty of Science, University of Bamenda, Cameroon; ^3^Department of Microbiology, Faculty of Science, University of Yaounde 1, Yaounde, Cameroon

## Abstract

The effects of chemicals commonly used in Cameroon to eliminate slime from the flesh of the African giant land snail, *Archachatina marginata*, during processing on some nutritional and biochemical parameters were investigated. Groups of snails were processed with these chemicals at three different concentrations. Proximate analysis of all the treated snail groups was carried out, and groups with the highest concentration of each chemical were used to compose diets for experimental rats. Thirty weanling male Wistar albino rats (31.25 ± 3.09 g) aged 21days old were distributed into four groups and fed with 10% protein based diets of *A. marginata* named D1 (washed with only water), D2 (lime C-treated), D3 (alum C-treated), and D4 (salt C-treated). The crude protein contents of the treated groups reduced significantly when compared with the control (CW), with lime C-treated (LC) having the least here and in crude fiber, but higher (LC, LB, and LA) in dry matter. There was a significant reduction in the crude lipid of alum C-treated (AC) and salt A-treated (SA). In vivo studies showed a general decrease in food consumption, weight gained, efficiency of feed utilization (EFU), true protein digestibility (TD) (except D2), and hematological indices (RBCs (red blood cells), PCV (packed cell volume) of the treated groups (D2, D3, D4) when compared to the control (D1). On the other hand, an increase in the relative weight of the liver (RWL), aspartate aminotransferase (AST), alanine aminotransferase (ALT), and total cholesterol was observed with some of the treated diets; meanwhile, protein efficiency ratio (PER), net protein ratio (NPR), relative weight of the kidneys (RWK), HDL cholesterol, and triglycerides were not affected by these diets. These chemicals should only be used at low concentrations or not at all because of its toxicity at high concentrations.

## 1. Introduction

Snails belong to a group of invertebrate animals classified under phylum mollusca [1]. They are the largest group under this phylum and are found worldwide in seas, freshwater, and in moist areas on land [2]. *A. marginata* is the most common specie of the African giant land snails having a morphology that is essentially divided into two parts, the body and the shell. The shell acts as its protective casing while the body is divided into three parts: the head, the foot, and the visceral mass [3–5]. The fleshy part of the body also known as the meat includes the head, foot, and reproductive organs (penis and oviduct) with a unique taste and aroma [5, 6]. A lot of research has been done on the nutritional value of *A. marginata* meat. Studies reveal that it contains a lot of proteins, minerals, and has low fats and cholesterol levels [5–9].

*A. marginata* meat is often processed with basic or acidic chemicals prior to consumption. It has been reported that antinutritional factors may be formed during food processing [10]. Alkaline processing or treatment induces two major chemical changes in proteins which are racemization of optically active amino acids and the formation of cross links such as lysinoalanine and lanthionine [11, 12]. These may lead to a reduction in digestibility and quality of proteins [13]. Also, acid treatment can result to deamination of amide nitrogen leading to the hydrolysis of peptide bonds, hence increased solubility of proteins, which may probably affect their digestibility [13, 14].

In Cameroon, especially in the South West Region, *A. marginata* meat commonly known as Nyamagoro or Congo meat or slow boys is consumed by almost all dwellers. It is used as a source of protein by these consumers and marketed in most regions of Cameroon. The flesh of these snails contains a lot of slime and when removed using the right method renders it more attractive and appetizing. Chemicals like lime, alum, and table salt are commonly used as desired by consumers during processing to get rid of the slime, which can still be removed with enough water but for the fact that it is time consuming as they think. Lime contains a lot of citric acid; Salt contains sodium chloride (NaCl) while alum is a class of double salt (K_2_SO_4_Al_2_[SO_4_]_3_.24H_2_O). These consumers are not aware of possible effects that these chemicals can cause when in contact with nutrients, the reason why these chemicals are used as desired. This investigation is of paramount importance to Cameroonians and the world at large, in order to create awareness thereby preventing public health challenges in the nearest future. There is no available scientific information on the effects of these chemicals on *A. marginata* meat as well as the bioavailability of nutrients when treated with these chemicals. This study was designed to evaluate the effects of using these chemicals at different concentrations when processing this meat on some nutritional and biochemical parameters.

## 2. Materials and Methods

### 2.1. Collection of Snail Samples

African giant land snails were purchased from Muyuka in the South West Region of Cameroon, and immediately transported in ventilated bags to the University of Dschang Snailery. On arrival, snail species such as *Achatina achatina* and *Achatina fulica* were separated from our specie of interest, *Archachatina marginata*, following the identification method described by Babalola and Akinsoyinu [9]. Mature *Archachatina marginata* of equal sizes was used for the study.

### 2.2. Collection of Chemicals Used for Processing Snails

Alum, table salt, and lime fruits were bought from a market in Dschang, West region of Cameroon.

### 2.3. Preparation of Raw Samples

The edible parts (meat) were carefully removed from the shells with the aid of a stainless iron hook and poured into stainless containers. The meat was weighed using an analytical mechanical balance into 10 groups of 2 kg each. The 10 groups were divided into one control group (massaged with no chemical (CW)) and nine test groups massaged as follows: three groups with table salt (at 1% (salt A), 2% (salt B), and 4% (salt C)): three others with alum (at 0.25% (alum A), 0.5% (alum B) and 1% (alum c)) and three with lime fruit (at 3.3% (lime A), 6.6% (lime B) and 13.2% (lime c)). Each group was analyzed in triplicates, giving a total of 30 samples from the 10 groups. Lime B (LB)-, alum B (AB)-, and salt B- (SB-) treated groups corresponded to the concentrations used by the consumers of *A. marginata* meat during processing. Based on this, we decided to divide and multiply these concentrations (lime B, alum B, salt B) by 2 to have the first (lime A, alum A, salt A) and third (lime C, alum C, salt C) sets of groups, respectively. The 10 groups were hand-massaged separately with their respective chemicals (test groups) or not (control group) for 2 minutes. The massaged groups were separately rinsed 4 times with 500 ml of clean tap water per rinsing process.

### 2.4. Preparation of Cooked Samples

The groups were separately boiled to dryness with 0.5 l of water for 25 minutes using a kitchen gas cooker (Arkays Double Burner Gas Stove, Model No JNY-TH2). The cooked groups were separately cooled, sliced into small pieces, and dehydrated in a moisture extractor oven at an average temperature of 60 ± 2°C for 12 hours. The cooked and dehydrated groups were separately ground using an electric blender (Moulinex Blender, Ref 0817896) to obtain meals which were stored for proximate analysis and formulation of test diets.

#### 2.4.1. Proximate Composition

The cooked, dehydrated, and ground samples (control, lime-treated, alum-treated and salt-treated) of *A. marginata* meat were separately analyzed for proximate composition. The A.O.A.C. [15] method was adopted for the estimation of ash, crude fiber, crude protein, and crude lipid. All analyses were carried out in triplicate.

#### 2.4.2. Preparation of Experimental Diets

Based on the results obtained from proximate analysis, we decided to continue with *A. marginata* meat groups that were treated with the highest concentration of chemicals (lime C, alum C, and salt C). This was simply because the effect of these chemicals was highly felt at these concentrations; as such, we wanted to equally know what could happen in vivo. The experimental diets were supplemented with cooked, dehydrated, and ground samples of control (CW), salt-treated (c), alum-treated (c) and lime-treated (c). A total of four experimental diets were prepared as per ICN [16] protocol. Diet 1 (D1) was composed with the control group, whereas diet 2 (D2), diet 3(D3), and diet 4 (D4) were composed with lime C-treated, alum C-treated, and salt C-treated groups, respectively. Diet 0 (D0) was the protein-free (PF) diet.  Table 1 gives the composition of the experimental diets. The four test diets (D1, D2, D3, and D4) were formulated on the basis of their proximate composition with all the diets containing 10% protein (w/w).

### 2.5. Experimental Design

A rat feeding study was carried out to determine the protein quality and digestibility of the test diets based on rat growth. Thirty (30) weanling male albino *Wistar* rats (31.25 ± 3.09 g) aged 21 days, bred in the Biochemistry Departmental Animal House, University of Dschang, Cameroon, were used. The animals (rats) were divided into 5 groups of six animals each. Difference in the average group weight was not more than 3 as recommended by A.O.A.C. [17]. The animal cages were designed as described by Sarwar and Estatira [18]. The animals were housed individually in stainless steel screening bottom plastic cages, to permit free dropping of feces and stainless steel mesh tops to ease ventilation. Highly absorbent paper was place under the cages to catch spilled food and to minimize the contamination of feces with urine. All the cages were placed away from direct sunlight in a cage rack near a window. They were thoroughly cleaned daily and maintained at room temperature with 12 hours light/dark cycle.

The different groups of rats were allocated to the different diets: group A was fed with D1 (control), group B with D2 (lime C-treated), group C with D3 (alum C-treated), group D with D4 (salt C-treated), and group E with D0 (protein free diet). The rats were given the corresponding diets and water ad libitum for 14 days, and records of daily food consumption and body weights were kept from the fifth day. The first 4 days were considered the acclimatization period. Daily records of the food consumed by rats were kept by weighing the food given and that which remained together with the amount of food spilled after 24 hours.

#### 2.5.1. Nutritional Evaluations

The efficiency of feed utilization (EFU), protein efficiency ratio (PER), net protein ratio (NPR), and true protein digestibility (TD) values (10 days) was also determined at the end of the experimental period [19]. The protein-free diet provided an estimate of metabolic fecal protein (MFP) needed for the calculation of TD.

*(1) Determination of Protein Digestibility*. The feces of each animal was collected every morning for the first 7 days after acclimatization. At the end of this period, the bulk feces for each rat was lyophilized, weighed, and ground. Records of daily food consumption and body weights were also kept during this collection period. The total nitrogen content of the ground feces was determined using the method of AOAC [20]. Six individual TD values per diet were then calculated using protein intake and fecal output data for each rat [19].

(1) $$\begin{array}{c} \text{TD }\left(\%\right)=\frac{\left[\text{PI}–\left(\text{FP}-\text{MFP}\right)\right]}{\text{PI}}\times 100, \end{array}$$

where PI is the protein intake, MFP is the metabolic fecal protein, and FP is the fecal protein.

(2) $$\begin{array}{c} \text{EFU}=\frac{\text{net weight gain }\left(\text{g}\right)\;}{\text{Total food eaten }\left(\text{g}\right)}\times 100\text{ g},\\ \text{EFU}=\frac{\text{netweightgain}\left(\text{g}\right)}{\text{Totalfoodeaten}\left(\text{g}\right)}\times 100\text{g},\\ \text{PER}=\frac{\text{weight gain of test animal}}{\text{Total protein consumed}},\\ \text{NPR}=\frac{\text{wt gain on test diets}+\text{wt loss on protein free diet }}{\text{Total test protein intake}}. \end{array}$$

#### 2.5.2. Biochemical Assay

*(1) Animal Sacrifice, Sample Collection and Analysis*. At the end of the experimental period, all the six animals in each group were anesthetized in chloroform vapor (one at a time), and blood was collected by cardiac puncture after dissection using 10 ml plastic syringes. About 6 ml of blood was transferred into heparinized tubes and nonheparinized tubes. The nonheparinized tubes were left for 3 hrs at room temperature to coagulate, after which they were centrifuged for 15 minutes at 3000 turns/min using a bench centrifuge to obtain serum. These sera were assayed for serum transaminases, i.e., alanine aminotransferase (ALT), aspartate aminotransferase (AST) [21], triglycerides [22], total cholesterol, and HDL cholesterol [23] using Kits from INMESCO. The heparinized blood was used for determination of some hematological parameters such as red blood cell count [24] and hematocrit evaluation [25].

Immediately after blood collection from the rats, organs like the liver and kidney were excised from the rats with the help of surgical forceps and scissors. They were blotted dry on a tissue paper and weighed. The weights of these organs were calculated:

(3) $$\begin{array}{c} \text{Relative organ weight}=\frac{\text{Organ weight }\left(\text{g}\right)\;}{\text{Body weight }\left(\text{g}\right)}\times 100. \end{array}$$

The risk factor of developing coronary heart diseases (CHD) was also calculated:

(4) $$\begin{array}{c} \text{Risk factor of CHD}=\frac{\text{serum LDL}-\text{cholesterol}}{\text{Serum HDL}-\text{cholesterol }}. \end{array}$$

### 2.6. Statistical Analysis

Analyzable data were subjected to the one-way analysis of variance (ANOVA), and differences between samples at *p* ≤ 0.05 were determined by the Waller-Duncan test using the Statistical Package for the Social Sciences (SPSS) version 11.0. The results were expressed (where appropriate) as mean ± standard deviation of the replicates.

## 3. Results and Discussion

### 3.1. Proximate Composition of Cooked, Dehydrated, and Ground Meat Samples

The proximate composition of the ten groups of cooked, dehydrated, and ground *A. marginata* meat processed with different chemicals at different concentrations is presented in Table 2.

The lime A-treated (LA) group had the highest dry matter (D.M) content while the lowest was recorded by the salt B-treated (SB) group when compared to the control (CW). Only groups treated with limes at different concentrations (LC, LB, and LA) were significantly higher (*p* < 0.05) than CW. On the other hand, the salt C-treated (SC) group recorded the highest ash content while the lime B-treated (LB) group had the lowest when compared with CW. Apart from group SC, other groups (alum C-treated (AC), alum A-treated (AA), and salt A-treated (SA)) equally had values for ash content significantly greater (*p* < 0.05) than CW. The crude protein contents of the treated groups reduced significantly (*p* < 0.05) when compared with the control (CW), with salt A-treated (SA) having the highest and lime C-treated (LC) the least. Also, LC recorded the least crude fiber content while LA had the highest when compared with CW; meanwhile, LB, SB, LA, and SA significantly increased (*p* < 0.05) when compared with the control (CW). There was a significant reduction (*p* < 0.05) in the crude lipid of alum C-treated (AC), and salt A-treated (SA) had the highest while the rest of the treated groups either increased or did not vary from CW.

The proximate composition analysis gives an idea of the nutrient content of a food. The results of this study revealed variation of nutrient values with samples. The reactions taking place in an environment of food (proteins) during processing determine its functionality and nutritional quality [10–12]. The least crude protein value recorded by lime C-treated sample was in line with the works of other researchers who demonstrated the treatment of protein sources with acids that increase solubility [14]. The ash content also followed a trend which was believed to be normal because dietary salt is rich in Na and Cl_2_, and alum is rich in K and S while lime does not contain minerals. The low crude fiber and crude fat contents were similar to earlier works carried out on snails, demonstrating their low lipid and crude fiber contents [5, 7, 26, 27].

### 3.2. Effects of Different Diets on the Performance of Rats

The weight gain, food consumption, and EFU of test diets evaluated after 10 days are shown in Table 3. There was a general decrease in food consumption, weight gained, and EFU of the treated groups (lime C-treated (D2), alum C-treated (D3), and salt C-treated (D4)) when compared to the control (D1). The decreased in food consumption and weight gain was significantly different (*p* < 0.05) in all the different test diets compared to D1. There was also a significant decrease (*p* < 0.05) in the EFU in D3 and D4 when compared to D1.

The different chemicals used in processing *A. marginata* meat might have induced varied effects on its palatability. In the case of lime C-treated diet (D2), the high food consumption recorded could be attributed to the sour taste, pleasant flavor, and aroma of lime fruit, coupled to it vitamin c content which is an appetizer [28]. Also, degradable reactions resulting from the environment of a food protein during processing may have contributed to undesirable flavor changes in the alum treated meat (D3) leading to low food consumption [10–12]. Weight gain was directly proportional to food consumption. The high weight gain observed with the lime C-treated suggests that there was better protein utilization in this group when compared with the other diets. The efficiency of the diets was decreased in the presence of these chemicals, with the exception of lime C-treated.

### 3.3. Effects of Different Diets on the Protein Quality of Rats

The PER, TD, and NPR evaluated after 10 days (Table 3) showed that the different diets had no effect on PER and NPR. The effect was only felt at the level of TD. Despite the general trend observed in TD, only the effect of D3 (alum C-treated) was significantly reduced (*p* < 0.05) when compared to the control (D1).

Digestibility indicates protein availability showing the amount of ingested protein that is hydrolyzed by digestive enzymes and absorbed by the body [29]. Studies have shown that the presence of certain antinutritional factors formed during food processing can substantially reduce protein digestibility in animals [10]. The TD of animals fed with D3 was significantly low (*p* < 0.05) indicating that certain antinutritional factors might have been formed during processing leading to a reduction in digestibility and quality of proteins [13]. The TD of animals fed with lime C-treated (D2) was higher than D1; this might be due to the presence of acids in this food from lime fruits which rendered it more available to the action of digestive enzymes at the level of the stomach, leading to an increased in digestibility.

### 3.4. Effects of Different Diets on Relative Liver/Kidney Weights and Hematological Indices of Rats

The relative liver and kidney weights, PCV, and RBC for rats fed with the test diets for 28 days (Table 4) showed that some of these biological parameters varied as a function of the treated diets. There was an increase in the relative weight of the liver except the alum C-treated group which was not significantly different from the control. There was no significant change in the relative weight of the kidneys, suggesting that the chemicals had no significant effects on them. The significant increase observed in lime C-treated and salt C-treated diets suggest that they are capable of inducing hypertrophy of the liver. This might be due to the presence of toxic substances in the prepared diets. The hepatic hyperplasia was further confirmed by the increased sera activities of ALT and AST in some cases. More so, the PCV values for all the test diets showed a significant decrease (*p* < 0.05) while there was a nonsignificant (*p* > 0.05) decrease in the RBC count when compared with the control (D1).

The estimation of the hematological indices of rats fed with the test diets gives an idea of the physiological condition of the blood and reticuloendothelial system [30]. Since a decrease in PCV can either be due to gastrointestinal bleeding, hemolysis, or nutritional defects (iron deficiency or vitamin B12) [31], the reduction in PCV might therefore be attributed to a suppressive action of the chemicals on erythropoiesis.

### 3.5. Effects of Different Diets on Serum Enzyme Activities of Rats

Table 4 gives the enzyme activities (ALT and AST) of sera determined after feeding the rats for 28 days. There was a significant increase (*p* < 0.05) in the AST activity of rats fed with the test diets when compared to the control (D1), with the highest recorded with the salt C-treated diet (D4). On the other hand, only the ALT activity of rats fed with lime C-treated diet (D2) increased significantly (*p* < 0.05) when compared with D1.

Alanine aminotransferase (ALT) and aspartate aminotransferase (AST) are transaminases concerned with amino acid metabolism. They are often used in clinical diagnosis to assess the integrity of certain body organs. Large amounts of AST are present in the liver, kidneys, heart, brain, pancreas, lungs, and skeletal muscle while ALT is found principally in the liver [32, 33]. Necrotic activities in these organs, especially the liver, will release abnormal quantities of these enzymes into the blood [34]. The high activities of both enzymes recorded in this study may reflect the severity of liver damage [32], since the serum or plasma levels of both AST and ALT rises whenever there is liver cell damage. The chemicals could affect the permeability of the cell membrane causing the membrane to become leaky, thus inducing the release of these enzymes from the cells into the blood stream [35] leading to high levels in serum as observed in the present study. This suggests that the chemicals may have significant cytotoxic effect on the liver.

### 3.6. Effects of Different Diets on Lipid Profile of Rats

The lipid profile of each group of rats evaluated post to the feeding experiment is presented in Table 5. Apart from total cholesterol that showed a significant increase (*p* < 0.05) in rats fed with D2 (lime C-treated) and D4 (salt C-treated), the other parameters (HDL cholesterol and triglycerides) did not show any significant (*p* > 0.05) variation when compared with D1 (control). This showed an increase in total cholesterol in rats induced by these diets. More so, the risk factors for developing CHD were generally low in all the diets when compared to other food sources.

The serum total cholesterol level was generally high in all the groups, demonstrating that, it can be influenced by dietary factor [36]. It is known that, plasma cholesterol is affected more by the level of dietary protein than by the type or level of dietary fat, the reason why lipogenesis is generally low in rats fed with high fat and low protein diet but high in a rich protein diet [37–39]. The lower the risks factor for CHD, the lower the risk of CHD development [40]. Thus, the low risk factors observed in the present work suggest that this meat could be recommended to those with coronary heart diseases (CHD) or those at risk of developing them.

## 4. Conclusion

The nutritional value of *A. marginata* meat decreases when processed with lime, alum, and salt in a concentration-dependent manner as well as it true digestibility. These chemicals are equally toxic and capable of inducing hypertrophy of the liver and can suppress erythropoiesis. As such, these chemicals should be used at very low concentration or not at all since the slime can still be removed without chemicals, but for the fact that it is time consuming. Thus, the use of just water in processing is recommended for all consumers.

## Figures and Tables

**Table 1:** Composition of the experimental diets used in the biological assay (g/100 g complete diet).

Ingredients (g/100 g)	Diets				
	D0	D1	D2	D3	D4
Corn starch	15	15	15	15	15
Corn oil	5	5	5	5	5
Mineral complex^b^	4	4	4	4	4
Vitamin complex^b^	1	1	1	1	1
Cellulose	7.9	7.9	7.9	7.9	7.9
Lime sample^a^	0	0	15.3	0	0
Alum sample^a^	0	0	0	15.1	0
Salt sample^a^	0	0	0	0	15.1
Control sample^a^	0	14.7	0	0	0
Sucrose	67.1	52.4	48.2	48	48
Total	100	100	100	100	100

D0: protein-free diet; D1: control (processed without chemicals); D2: processed with lime-C; D3: processed with alum-C; D4: processed with salt-C. ^a^cooked and dehydrated samples, ^b^supplied by clab Laboratory, Bafoussam- Cameroon. The crude proteins obtained from proximate analysis were converted to grams by making use of DM after which the amount of protein present in 10 g was determined by simple proportion.

**Table 2:** Proximate composition of the different samples of *Archachatina marginata* meat.

Groups	DM (%)	Ash (% DM)	Crude protein (% DM)	Crude fibre (% DM)	Lipid (% DM)
Control (CW)	93.12 ± 0.93^bc^	4.17 ± 0.50^b^	73.26 ± 0.50^e^	0.32 ± 0.02^b^	5.04 ± 0.75^b^
Lime 13.2% (LC)	95.02 ± 1.39^de^	4.71 ± 0.40^bc^	68.71 ± 0.50^a^	0.11 ± 0.01^a^	5.21 ± 0.69^b^
Alum 1% (AC)	94.10 ± 0.51^cd^	4.98 ± 0.34^cd^	70.19 ± 0.40^b^	0.42 ± 0.07^bc^	3.66 ± 0.50^a^
Salt 4% (SC)	92.62 ± 0.50^bc^	7.09 ± 0.72^e^	71.67 ± 0.47^c^	0.32 ± 0.05^b^	5.06 ± 0.70^b^
Lime 6.6% (LB)	94.71 ± o.49^d^	3.31 ± 0.10^a^	69.05 ± 0.50^a^	0.69 ± 0.20^de^	5.18 ± 0.78^b^
Alum 0.5% (AB)	93.75 ± 1.02^bcd^	4.49 ± 0.33^bc^	70.40 ± 0.51^b^	0.22 ± 0.09^ab^	5.22 ± 0.81^b^
Salt 2% (SB)	90.94 ± 0.20^a^	4.66 ± 0.38^bc^	72.00 ± 0.30^cd^	0.56 ± 0.18^cd^	4.71 ± 0.28^ab^
Lime 3.3% (LA)	96.50 ± 1.48^e^	4.14 ± 0.20^b^	70.02 ± 0.48^b^	0.820 ± 0.20^e^	4.76 ± 0.50^ab^
Alum 0.25 (AA)	93.62 ± 1.10^bcd^	4.86 ± 0.11^cd^	71.74 ± 0.55^c^	0.32 ± 0.03^b^	6.59 ± 1.00^c^
Salt 1% (SA)	92.46 ± 0.50^b^	5.47 ± 0.52^d^	72.50 ± 0.60^d^	0.54 ± 0.18^cd^	6.80 ± 0.92^c^

Values are mean ± SD of 3 determinants, sample size (*n*) = 30. Along the columns, values with the same letter (a, b, c, d, e) are not significantly different (*p* > 0.05). D.M: dry matter; CW: control sample washed without chemicals; LC: lime-treated sample at 13.2%; AC: alum-treated sample at 1%; SC: salt-treated sample at 4%; LB: lime-treated sample at 6.6%; AB: alum-treated sample at 0.5%; SB: salt-treated sample at 2%; LA: lime-treated sample at 3.3%; AA: alum-treated sample at 0.25%; SA: salt-treated sample at 1%.

**Table 3:** Effects of different treated diets on the performance of rats and protein quality.

Parameters	Diets			
	D1	D2	D3	D4
Food consumed (g)	114.83 ± 1.86^a^	78.40 ± 1.04^b^	54.90 ± 1.61^d^	69.20 ± 1.59^c^
Weight gained (g)	31.31 ± 0.99^a^	21.38 ± 1.08^b^	13.60 ± 1.06^d^	16.90 ± 1.29^c^
EFU (g/100 g)	27.26 ± 1.03^b^	27.27 ± 1.47^b^	24.77 ± 1.16^a^	24.42 ± 2.16^a^
PER	2.60 ± 0.61^a^	2.70 ± 0.19^a^	2.40 ± 0.43^a^	2.50 ± 0.31^a^
NPR (g)	3.00 ± 0.58^a^	3.23 ± 0.18^a^	2.70 ± 0.58^a^	2.80 ± 0.11^a^
TD (%)	76.70 ± 2.76^bc^	78.20 ± 2.71^c^	70.00 ± 3.98^a^	74.19 ± 0.22^ab^

Values are mean ± SD of 6 determinants, sample size (*n*) = 30. Along the columns, values with the same letter (a, b, c, d) are not significantly different (*p* > 0.05). D1: control (washed without chemicals); D2: processed with lime-C; D3: processed with alum-C; D4: processed with salt-C; EFU: efficiency of feed utilization; PER: protein efficiency ratio; T.D: true protein digestibility; NPR: net protein ratio.

**Table 4:** Effect of the different treated diets on the biological parameters and serum enzyme activities of rats.

Parameters	Diets			
	D1	D2	D3	D4
RWL (%)	4.30 ± 0.34^a^	4.80 ± 0.43^b^	4.10 ± 0.21^a^	5.10 ± 0.13^b^
RWK (%)	1.06 ± 0.33^a^	1.00 ± 0.21^a^	1.00 ± 0.13^a^	0.90 ± 0.13^a^
PCV (%)	43.53 ± 1.54^b^	38.20 ± 1.19^a^	38.28 ± 0.98^a^	36.70 ± 1.58^a^
RBC/mm^3^×106)	7.17 ± 0.55^a^	6.80 ± 0.79^a^	6.90 ± 0.82^a^	6.60 ± 0.83^a^
ALT (U/L)	5.46 ± 0.83^a^	8.44 ± 2.02^b^	4.60 ± 0.84^a^	4.79 ± 0.51^a^
AST (U/L)	7.27 ± 0.50^a^	8.14 ± 0.50^ab^	9.16 ± 1.85^b^	12.51 ± 1.32^c^

Values are mean ± SD of 6 determinants, sample size (*n*) = 24. Along the columns, values with the same letter (a, b) are not significantly different (*p* > 0.05). D1: control (washed without chemicals); D2: processed with lime-C; D3: processed with alum-C; D4: processed with salt-C; RWL: relative weight of the liver; RWK: relative weight of the kidney; PCV: packed cell volume; RBC: red blood cell; ALT: alanine aminotransferase; AST: aspartate aminotransferase.

**Table 5:** Effects of treated diets on lipid profile of rats.

Parameters	Diets			
	D1	D2	D3	D4
Total cholesterol (activity (mmol/L)	4.67 ± 0.13^a^	7.34 ± 1.41^b^	4.79 ± 0.16^a^	7.47 ± 0.28^b^
HDL cholesterol (activity (mmol/L)	3.21 ± 0.30^a^	3.15 ± 1.12^a^	3.47 ± 1.18^a^	3.29 ± 0.47^a^
Triglycerides (activity (mmol/L))	0.92 ± 0.43^a^	0.74 ± 0.21^a^	1.17 ± 0.51^a^	0.81 ± 0.57^a^
LDL cholesterol (activity (mmol/L))	0.11 ± 0.02^a^	0.69 ± 0.12^b^	0.03 ± 0.00^a^	0.67 ± 0.02^b^
VLDL+LDL cholesterol (activity (mmol/L)	1.46 ± 0.11^a^	4.19 ± 0.35^b^	1.32 ± 0.30^a^	4.18 ± 0.09^b^
Risk factor for CHD	0.03 ± 0.00^a^	0.22 ± 0.01^b^	0.01 ± 0.00^a^	0.20 ± 0.00^b^

Values are mean ± SD of 6 determinants, sample size (*n*) = 24. Along the columns, values with the same letter (a, b) are not significantly different (*p* > 0.05). D1: control (washed without chemicals); D2: processed with lime-C; D3: processed with alum-C; D4: processed with salt-C; HDL: high-density lipoprotein; LDL: low-density lipoprotein; VLDL: very low-density lipoprotein; CHD: coronary heart diseases.

## Data Availability

The [data type] data used to support the findings of this study are included within the article.
